# Acute heart failure as an atypical presentation of Takayasu arteritis: The value of multi‐modality imaging

**DOI:** 10.1002/ccr3.5306

**Published:** 2022-01-25

**Authors:** Sepideh Djafari Naeini, Fariba Bayat, Golnaz Houshmand

**Affiliations:** ^1^ Cardiovascular Research Centre Shahid Beheshti University of Medical Sciences Tehran Iran; ^2^ Cardiovascular Research Centre Shahid Beheshti University of Medical Sciences Tehran Iran; ^3^ Rajaie Cardiovascular, Medical and Research Centre Iran University of Medical Sciences Tehran Iran

**Keywords:** cardiac magnetic resonance, echocardiography, myocarditis, Takayasu, valvular heart disease, vasculitis

## Abstract

Takayasu arteritis (TA) is an inflammatory disease that affects the aorta and the major branch arteries. Here, we describe an atypical presentation of the disease with heart failure.

## Graphical abstract

Multi‐modality imaging, particularly the use of cardiac magnetic resonance, is considerably helpful in diagnosing and managing Takayasu arteritis (TA). In the workup of new‐onset heart failure in young patients employing cardiac MRI can be promising in making the proper diagnosis and subsequent treatment.

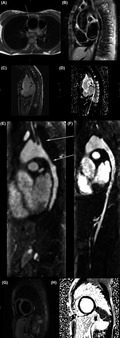

## 1 CASE PRESENTATION

A 23‐year‐old woman was admitted to the hospital due to dyspnea at rest. She had been suffering from exertional dyspnea (New York Heart Association Class 2 to 3) for a month. She had no previous history of a medical condition or habitual history other than occasional smoking. She denied any allergic history or other symptoms such as claudication, oral ulcer, fever, or joint pain.

On physical examination, the heart rate was 102 beats/min, the respiratory rate was 17/min, oxygen saturation at room air was 91%, and the oral temperature was 37°C. She had hardly palpable radial and dorsal pedis pulses with a low blood pressure of about 90/60 mmHg (bilateral brachial with up to 10 mmHg difference). Bilateral basilar rales were present in lung examination. A decrescendo early‐diastolic blowing murmur (2/6) in the 3rd intercostal space was heard in heart auscultation. A harsh bruit was heard in both carotids, subclavian, renal, and iliac arteries.

**FIGURE 1:**
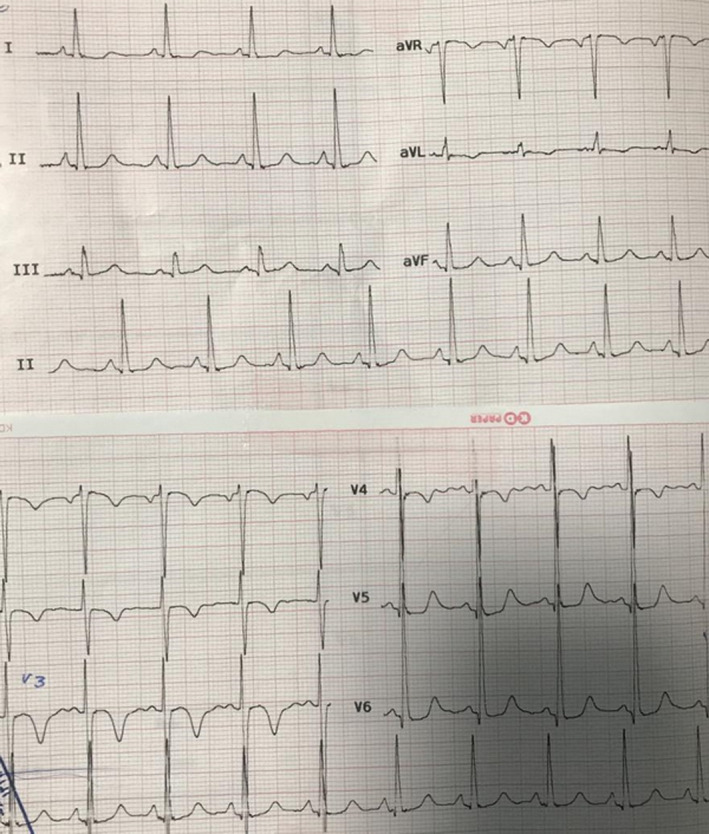
12‐lead ECG showing high voltage QRS in all leads with ST depression and strain pattern in I‐II‐V5‐V6

12‐lead electrocardiography (ECG) revealed evidence of left ventricular hypertrophy (Figure 1).

Laboratory tests showed a high erythrocyte sedimentation rate (ESR) of 44 mm/h (0–15 mm/h normal value), leukocytosis with white blood cell count of 12 × 10^9^/ml (4.5–11 × 10^9^/L normal level), high N‐terminal pro B‐type natriuretic peptide (NT‐pro‐BNP) level of 7700 pg/ml (<120 pg/ml normal level), troponin I level of 0.02 ng/ml (normal <0.03 ng/ml) with normal thyroid, renal and hepatic function. The polymerase chain reaction test of the nasopharyngeal swab for SARS‐‐CoV‐2 was negative.

**FIGURE 2:**
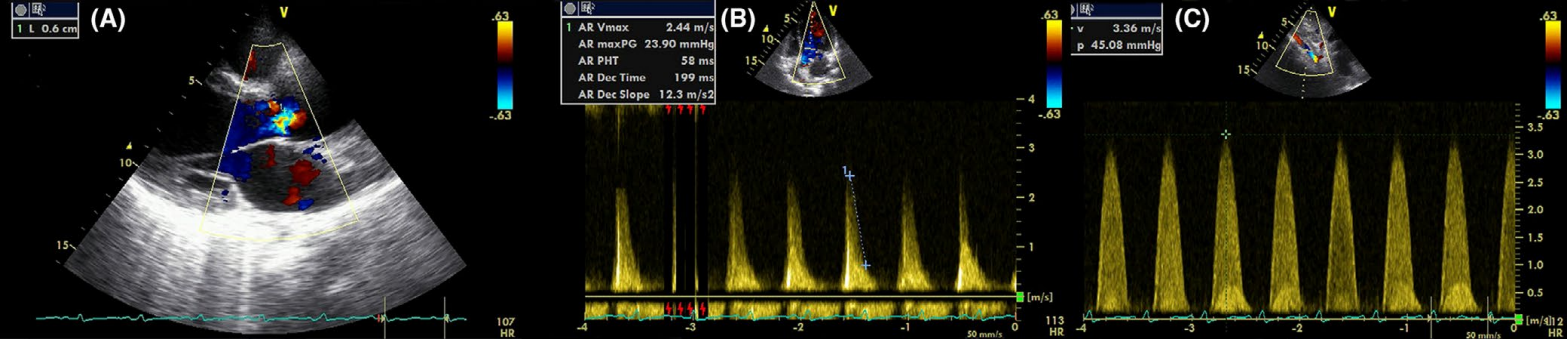
(A) Color Doppler image at parasternal long axis showing vena contracta of 6 mm aortic regurgitation (B) continuous‐wave Doppler of aortic regurgitation showing short pressure half time of 58 ms (C) Continuous‐wave Doppler echo showing increased velocity 3 m/s at the abdominal level

Transthoracic echocardiography (TTE) revealed left ventricular (LV) enlargement with severe LV systolic dysfunction, trileaflet aortic valve with severe aortic regurgitation (AR; vena contracta 6 mm, pressure half time 58 ms; Figure 2A,B) and dilated ascending aorta. The right ventricle was normal and pulmonary artery pressure was 42 mmHg. There was diffuse narrowing in the descending thoracic aorta and abdominal aorta with significant stenosis detected in the distal segment of the abdominal aorta with a high systolic peak gradient(48 mmhg) in the Doppler study by TTE (Figure 2C). The patient was referred for CMR for further evaluation.

**FIGURE 3:**
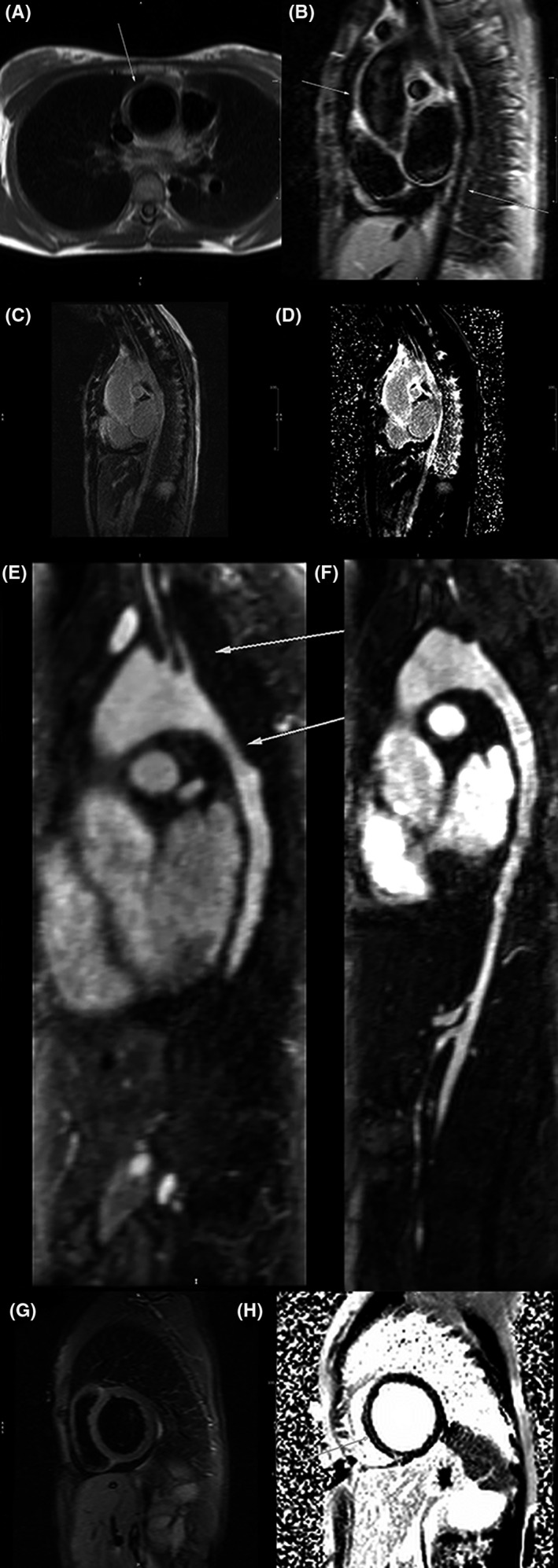
(A) T1 weighted axial image showed increased aortic wall thickness (B) T2 weighted fat‐saturated candy cane view of aorta revealed high signal intensity of aortic wall (C, D) Magnitude and phase‐sensitive inversion recovery late gadolinium images showing enhancement of whole thoracic aortic wall (E, F) Magnetic resonance angiography multiplanar reconstruction showing multiple stenoses of the left common carotid and left subclavian artery and proximal descending aorta. (G, H) T2 weighted fat‐saturated and late gadolinium sequences in short axis showing enhancement of the mid septal wall

Cardiac magnetic resonance (CMR) showed a severely enlarged left ventricle (LV end‐diastolic volume index 138 ml/m^2^) with severely reduced LV ejection fraction (LVEF 34%). Severe aortic regurgitation (anatomic regurgitant orifice measuring 0.35 cm^2^ and regurgitant fraction 33%) was confirmed (Video S1 and S2). The aortic annulus measured 27*18 cm, the sinus diameter was 38 mm, the sinotubular junction was 36 mm, and the dilated ascending aorta at the level of the right pulmonary artery measured 40 mm. T1 weighted turbo spin‐echo images showed increased aortic wall thickness.T2 weighted fat‐saturated and late gadolinium sequences revealed diffuse enhancement in the thoracic aortic wall, all in favor of active inflammation of the aorta (Figure 3A‐D). Magnetic resonance angiography (MRA) showed diffuse stenosis at multiple levels in the mid arch, arch branches, and descending aorta and the branches (Figure 3E,F). In the myocardium, there was an enhancement in the septal wall in the T2 weighted fat‐saturated with correspondent enhancement in the late gadolinium images, which were compatible with active myocarditis^1^ (Figure 3G, H).

The criteria for diagnosis of Takayasu arteritis (TA) were met in this young woman based on the presence of bruit in the aortic arch arteries, reduced pulses, stenosis in the aortic arch branches, descending aorta, and its branches in MR angiography.^2^ Having diagnosed active disease in the inflammatory phase with concomitant cardiac involvement, induction therapy with a corticosteroid (intravenous methylprednisolone 1 mg/Kg) for 3 days with adjunct immunosuppressive therapy with mycophenolate mofetil (MMF) were added to the initial heart failure treatment (ie, enalapril, spironolactone, and furosemide) which resulted in clinical and echocardiographic improvement. LVEF improved to 45% in 1‐month follow‐up TTE with no change in AR severity. The patient is currently under close follow‐up without any dyspnea or other heart failure‐related symptoms.

## 2 DISCUSSION

We presented a case of a young woman with acute failure. Physical examination was given us clues of bruit on large branches. The laboratory findings were in favor of inflammation, and echocardiography showed us enlarged left ventricular, severe aortic regurgitation with high gradient detected in descending aorta. Cardiac MRI revealed myocardial and aortic wall inflammation with narrowing of major branches of the aortic arch and descending aorta. Takayasu arteritis (TA) can be diagnosed utilizing an integrated method that incorporates multi‐modality cardiac imaging. This allowed us to thoroughly evaluate the cardiovascular complication of this multi‐faceted disease and select the appropriate treatment.

Takayasu arteritis is a granulomatous, chronic large‐vessel pan‐arteritis that mostly affects the aorta and its main branch arteries, although it can also cause cardiac and valve involvement. Heart failure as the first presentation of the TA is rare but has been reported.^3^, ^4^, ^5^, ^6^

As a large‐vessel vasculitis with unknown etiology, TA predominantly affects young Asian women but can be seen in different parts of the world.^4^ The entire aorta and major aortic branches can be involved; however, the distribution of involvement varies in each part of the world.^7^ TA has a highly variable presentation, including constitutional symptoms, valvular insufficiency, LV dysfunction and thrombus formation in ventricles and pulmonary artery, are reported among the first presentations.^3^, ^7^, ^8^ Coronary artery disease, pulmonary hypertension, or hypertrophied left ventricle are the other possible presentations.^8^ Atypical forms of initial presentation may lead to a delay in diagnosis and alter the outcome. Aortic regurgitation and renovascular‐induced hypertension have been introduced as the known causes of symptomatic myocardial damage.^3^ The prevalence of aortic regurgitation in TA is reported to be as high as 25%, with a significant prognostic impact.^4^ In pathological studies on post‐surgical specimens, thickening of intima, media, and adventitia of aortic wall has been documented. AR is usually due to annular dilatation.^4^ In our patient, a combination of annular and sinotubular junction dilatation and valvulitis is the probable cause of AR based on echocardiographic and CMR studies. Surgical intervention of AR in this context has specific limitations because of aortic fragility and inflammation but seems to have acceptable results in experienced centers.^4^ Myocarditis may present in the inflammatory phase of the disease. Inflammatory cell infiltration and granuloma formation followed by sclerosis have a major role in the pathophysiology of the disease.^9^

Endomyocardial biopsy is a golden standard for detecting myocarditis, but it is invasive with potential procedural complications. Detecting active inflammation is sometimes challenging and can be assessed through a combination of symptoms, and laboratory and imaging findings. Cardiac magnetic resonance imaging (CMR) is a comprehensive imaging technique that offers evaluating exact cardiac size and function, valvular function, identifies myocardial inflammation, and fibrosis and depicts the major arteries' walls and main branches..^10^ CMR may not be available in all contexts; nevertheless, its availability and expertise are growing considering its advantages in measuring function, anatomy, and tissue characterization. The CMR's high specificity and sensitivity in detecting myocardial inflammation make it the recommended modality in modern cardiology practice as a non‐invasive alternative to endomyocardial biopsy in new‐onset heart failure and in patients with suspected myocarditis both in the initial and during follow‐up phases.^11^ Our patient had clues of large‐vessel vasculitis accompanied by heart failure in physical examination and initial echocardiography. In this case, using CMR allowed us to diagnosis, see the whole picture of the disease and assess disease activity and severity. Positron emission tomography (PET) is another useful method to assess the presence of inflammation with some limitations with regarding the availability and the cost.^7^

The presence of active inflammation in the myocardium and aorta in our patients demonstrated a chronic and persistent inflammatory process that requires intensive anti‐inflammatory treatment. Corticosteroids are usually the primary induction therapy in TA. Other immunosuppressive regimens (ie, mycophenolate mofetil, azathioprine, and methotrexate) are commonly used as adjunctive therapy in maintenance.^7^

## 3 CONCLUSION

Takayasu arteritis is a rare rheumatologic disorder with various initial presentations; we described heart failure symptomes as the first presentation of this disease. Multi‐modality imaging assisted us in making the diagnosis by providing a full assessment of this multi‐faceted disease and in determining treatment choices and follow‐up care. Early treatment strategy with an immunosuppressive regimen may result in rapid recovery and better outcomes.

## CONFLICT OF INTEREST

The authors declare that there is no conflict of interest.

## AUTHOR CONTRIBUTIONS

SDN contributed to the management and follow‐up of the patient and writing the manuscript. FB contributed to reading the echocardiography and reviewing the manuscript. GH contributed to reading the cardiac MRI, writing the manuscript, and reviewing the manuscript.

## ETHICAL APPROVAL

The patient signed the informed consent modules and accepted the publication of clinical data for research and scientific purposes.

## CONSENT

Written informed consent was obtained from the patient to publish this report in accordance with the journal's patient consent policy.

## Supporting information

Video S1

Video S2

Supplementary Material

## Data Availability

The data of this article will be shared on request.
