# Supplementary material for: Acute heart failure as an atypical presentation of Takayasu arteritis: The value of multi‐modality imaging
Source: Clin Case Rep. 2022 Jan 25;10(1):e05306. doi: 10.1002/ccr3.5306 (PMC8787727; doi:10.1002/ccr3.5306)
Supplement: Supplementary file 3 — Supplementary Material [file CCR3-10-e05306-s001.docx]

**VIDEO 1** CMR cine sequence 3chamber showing significant aortic regurgitation

**VIDEO 2** Flow velocity mapping sequence in-plane flow of candy cane aortic view showing aliasing of velocity in the mid-thoracic aorta
